# Dietary approaches to stop hypertension (DASH) score and obesity phenotypes in children and adolescents

**DOI:** 10.1186/s12937-020-00631-y

**Published:** 2020-10-04

**Authors:** Hamed Rahimi, Emad Yuzbashian, Rahim Zareie, Golaleh Asghari, Abolghassem Djazayery, Ariyo Movahedi, Parvin Mirmiran

**Affiliations:** 1 Nutrition and Endocrine Research Centre, Research Institute for Endocrine Sciences, Shahid Beheshti University of Medical Sciences, P.O. Box: 19395-4763, Tehran, 1985717413 Iran; 2 Department of Nutrition, Science and Research Branch, Islamic Azad University, Tehran, Iran

**Keywords:** Paediatric, Adolescence, Obesity phenotype, Cardiometabolic risk factors, Dietary approaches to stop hypertension

## Abstract

**Background:**

The prevalence of obesity and its two important phenotypes, the metabolically healthy obese (MHO) and the metabolically unhealthy obese (MUO) are 10.9, 9.1, and 1.8%, respectively, among children and adolescents in Iran. Data on the link between diet quality indices and obesity phenotypes in children and adolescents is scarce. The present study aimed to assess the association of the Dietary Approaches to Stop Hypertension (DASH) score with MHO and MUO, as well as with cardiometabolic risk factors (RFs) in children and adolescents with excess weight.

**Methods:**

This cross-sectional study was conducted on 341 children and adolescents with excess weight aged 6–13 years, selected from primary schools of Tehran. The DASH score was determined based on eight components using a valid and reliable food frequency questionnaire. Anthropometric measures, insulin, fasting plasma glucose, lipid profile, and physical activity levels were collected. MUO was classified based on two definitions: having 2 or more cardiometabolic RFs, or being insulin resistant determined by a homeostatic model assessment of insulin resistance (HOMA-IR) ≥ 3.16. Multivariable logistic regression models were used to estimate the odds ratios (ORs) and 95% confidence intervals (CIs) for MUO phenotypes and cardiometabolic RFs in each tertile of the DASH score after adjustment for confounders.

**Results:**

The mean ± SD for age and DASH score was 9.3 ± 1.7 years and 24.0 ± 4.9, respectively. The prevalence of MUO was 62.2% based on RFs, and 43.4% based on HOMA-IR. Participants in the highest tertile of the DASH score had significantly decreased odds for MUO based on HOMA-IR (OR = 0.49; 95% CI: 0.28–0.87) compared with those in the lowest tertile, after adjustment for confounders. However, there were no associations between the DASH score and any of cardiometabolic RFs, or MUO based on RFs (OR = 0.68; 95% CI: 0.38–1.20).

**Conclusion:**

The DASH score was inversely associated with MUO based on HOMA-IR, but not associated with MUO based on cardiometabolic RFs in this sample of children and adolescents. A DASH-style diet may have favourable effects on insulin sensitivity among children and adolescents with excess weight. Universal definitions for MHO/MUO are required, and longitudinal studies recommended to shed light upon this subject.

## Background

Overweight and obesity are complex and heterogeneous conditions with phenotypic variations. Two phenotypes, which have drawn much attention recently, are the metabolically “healthy” overweight/obese (MHO) and the metabolically “unhealthy” overweight/obese (MUO). MHO individuals have more favourable lipid profiles, higher insulin sensitivity, and lower risks for cardiovascular disease (CVD) during childhood, adolescence, and adulthood, compared to their MUO counterparts [1, 2]. Interestingly, individuals who have had the MHO phenotype in childhood show a better cardiometabolic profile later in adulthood compared to those who have had the MUO phenotype [3]. It has been suggested that paediatric overweight and obesity, compared to paediatric metabolic syndrome (MetS), is a better predictor of adulthood cardiometabolic risk factors (RFs) and MetS [4, 5]. This further highlights the importance of obesity phenotypes in children and adolescents, since MHO and MUO comprise both excess weight and cardiometabolic RFs. A recent study among children and adolescents in Iran reported the prevalence of obesity, MHO, and MUO as 10.9, 9.1, and 1.8%, respectively [6]. Meanwhile, in the US, approximately 65% of adolescents affected by obesity have the MHO phenotype, while only 32% of adults with excess weight have this phenotype. Thus, a shift of cardiometabolic risk from MHO towards the “unhealthy” phenotype appears to occur during the transition from childhood to adulthood [7]. Although there is no universal definition for obesity phenotypes, the importance of cardiometabolic RFs and the presence of insulin resistance (IR) in obesity makes them the most prevalent definitions for MHO and MUO [8].

Lifestyle factors, including dietary patterns and physical activity, are the major factors that could affect obesity phenotypes [9], and possibly underlie the shift from MHO towards MUO later in life. Iran and most other developing countries in the Middle East and North Africa are going through a transition period in terms of nutrition and lifestyle, including a more Westernised dietary pattern characterised by higher energy, fast and processed food, and fat intakes, as well as physical inactivity, all of which have resulted in a higher prevalence of obesity, cardiometabolic RFs, and non-communicable diseases [10]. Various diet quality indices have been developed to assess adherence to desirable priori-defined diets and patterns comprehensively and to investigate the health effects of these diets. Of the priori-defined dietary patterns, the Dietary Approaches to Stop Hypertension (DASH) diet, which recommends higher intakes of whole grains, fruits, vegetables, nuts, seeds, legumes, and low-fat dairy, and lower intakes of processed meat, sodium, and sweetened beverages, was originally developed to manage high blood pressure [11]. The DASH score has previously been studied in children and adolescents in Iran, with higher scores being inversely associated with MetS, hypertension, hyperglycaemia, and obesity [12, 13]. However, no studies in children and adolescents have directly investigated the association between the DASH score and obesity phenotypes, while the studies in adults showing favourable associations with the metabolically healthy phenotypes [14, 15].

Considering the nutritional transition and the prevalence of paediatric obesity in Iran, as well as the transition towards the unhealthy phenotype which seems to occur with age, assessing dietary factors associated with paediatric obesity phenotypes could provide useful data for the prevention of non-communicable diseases. Moreover, the favourable effects of a DASH-style diet on cardiometabolic RFs and metabolic health could make it a viable option for assessing diet quality. Therefore, we aimed to investigate the association of the DASH score with obesity phenotypes, as well as with cardiometabolic RFs among children and adolescents with excess weight aged 6–13 years.

## Methods

### Study population

The current cross-sectional study was carried out on 378 children and adolescents affected by overweight and obesity (body mass index (BMI) Z-score ≥ 1 according to age- and sex-specific World Health Organization criteria [16]) aged 6–13 years. The sample size was calculated based on a previous study of the DASH score and components of MetS (TG), with a standard deviation of 4.3 for the DASH score and 55 mg/dL for TG [12, 17]. Considering α = 0.5, power = 0.995, design effect = 1.2, and standard deviation of those variables, the minimum sample size required was 322 individuals. Participants were randomly selected from students with excess weight studying in primary schools located in three main districts of Tehran, Iran, using random number tables to select schools and students. Individuals were deemed eligible for inclusion if they were not on special diets, had no diagnosed illnesses such as diabetes, liver, or kidney diseases, and were not taking any pharmaceutical agents that affect glucose and lipid metabolism or any dietary supplements. Participants with incomplete dietary data (*n* = 12), and those who over- or under-reported their dietary intake were excluded (*n* = 16). To define over- and under-reports, the energy intake was divided by the estimated energy requirement according to equations proposed by the Institute of Medicine [18]; those not within the ±2SD range were deemed over- and under-reports. Additionally, subjects who had key missing biochemical or anthropometric values were also excluded (*n* = 9). Finally, statistical analyses were performed on 341 participants.

Written informed consent was obtained from children’s parents or legal guardians. Protocols of the study were approved by the institutional ethics committee of the Research Institute for Endocrine Sciences, Shahid Beheshti University of Medical Sciences (Ethics approval ID: IR.SBMU.ENDOCRINE.REC.1395.373).

### Anthropometrics, biochemical assay, and other measurements

Trained nutritionists with high experience in the paediatric field recorded anthropometric measurements according to standard methods. Weight was recorded to the nearest 0.1 kg while subjects were barefoot and in light clothing, using the scale function of GAIA 359 PLUS body composition analyser (Jawon Medical Co. Ltd., Shinsang, Korea). Height was measured to the nearest 0.5 cm, while standing with shoulders in normal alignment and barefoot, using a stadiometer. BMI was calculated as weight (in kilograms) divided by height (in meters) squared (kg/m^2^). Waist circumference (WC) was measured to the nearest 0.5 cm using a nonelastic tape, midway between the iliac crest and the lowest rib, after gentle respiration in standing position, and without any pressure to the body surface. Arterial blood pressure was measured manually on the right arm using a mercury sphygmomanometer with a suitable cuff size after 15 min of rest, by the Korotkoff sound technique. Systolic blood pressure (SBP) was determined by the onset of the tapping sound, and diastolic blood pressure (DBP) was determined at the disappearance of the sound. It was measured twice, at least one minute apart, with the average considered as the subject’s blood pressure.

Information on physical activity was collected using the Modifiable Activity Questionnaire (MAQ), to calculate metabolic equivalent task (MET) hours per week; high reliability (97%) and moderate validity (49%) have previously been ascertained for the Persian translated MAQ in adolescents [19].

Blood samples were drawn between 7:00 and 9:00 AM, after 12–14 h of overnight fasting, and centrifuged within 30–45 min of collection and analysed on the same day. Fasting plasma glucose (FPG) and serum triglycerides (TG) concentrations were measured by the enzymatic colorimetric method, using glucose oxidase and glycerol phosphate oxidase, respectively. High-density lipoprotein cholesterol (HDL-C) was measured after precipitation of the apolipoprotein B-containing lipoproteins with phosphotungstic acid. All kits were provided by Pars Azmoon, Tehran, Iran, and adapted to a Selectra auto analyser (Vital Scientific, Spankeren, The Netherlands). Inter- and intra-assay coefficients of variation (CV) were 1.1 and 1.4% for FPG, 1.1 and 3.1% for HDL-C, and 1.5 and 3.7% for TG, respectively.

Fasting serum insulin was determined by the electrochemiluminescence immunoassay (ECLIA) method, using Roche Diagnostics kits and the Roche/Hitachi Cobas e-411 analyser (Roche Diagnostics, GmbH, Mannheim, Germany). Intra- and inter-assay CV were 1.3 and 2.5%, respectively.

Pubertal status was assessed by a paediatric endocrinologist and according to Tanner stages, dividing the participants into the following two groups based on breast and genital growth stages: pre-pubertal (boys at genital stage I, girls at breast stage I) and pubertal (boys at genital stage ≥II, girls at breast stage ≥II).

### Dietary assessment

Regular dietary intakes of the participants were assessed in face-to-face interviews by trained dietitians, using a valid and reliable semi-quantitative food frequency questionnaire (FFQ). The validity and reliability of the FFQ have previously been reported [20, 21]. Participants were asked to designate how frequently they consumed each food item during the previous year on a daily, weekly, or monthly basis. The US Department of Agriculture (USDA) serving sizes were specified for each food item on the FFQ whenever possible; otherwise, household measures were reported and then converted to grams and servings. In case children had difficulty recalling, mothers were asked about the type and quantity of meals and snacks. Since the Iranian Food Composition Table (FCT) is incomplete, the USDA FCT was used [22]; for special or traditional foods not listed in the USDA FCT, the Iranian FCT was used alternatively [23].

Iranian diet contains varying amounts of grains, legumes, plant and animal protein, vegetables, fruits, dairy products, nuts and seeds, fast and processed food, sweetened beverages, and sodium [24], all of which are evaluated in the DASH score. Moreover, previous paediatric studies in Iran have been able to observe associations between the DASH score and cardiometabolic RFs [12, 13]. Taking these facts into account, the DASH score was deemed a relevant and practical diet quality index in the context of the Iranian diet. Initially, to neutralise the effect of energy intakes on the eight components of the DASH score, each food group intake was calculated per 1000 kcal. Then the DASH score was computed according to Fung et al. [25]. In short, the DASH score rewards points for high intakes of the five food groups, including fruits, vegetables, nuts and seeds and legumes, low-fat dairy products, and whole grains according to quintile rankings (i.e., participants in the lowest quintiles receive 1 point, those in the 2nd, 3rd, and 4th quintiles receive 2, 3, and 4 points respectively, and the highest quintiles, 5 points). Regarding the intakes of sodium, sweetened beverages, and red and processed meat, which are minimised in the DASH diet, participants in the lower quintiles of intakes scored higher points (i.e., the lowest quintiles are assigned 5 points and the highest quintiles, 1 point). We then summed up the eight component scores to obtain the overall DASH score of a participant, ranging from 8 to 40.

### Definitions

Overweight was defined as being between 1SD and 2SD, and obesity as being above 2SD in sex-specific BMI-for-age (5–19 years) charts of the World Health Organization [16].

Two definitions were used to categorise MHO/MUO. The first one was based on metabolic RFs, in which MUO was defined as having two or more of the following cardiometabolic abnormalities: WC ≥90th percentile for age and sex according to national reference curves [26]; SBP and DBP ≥90th percentile for sex, age, and height based on the National Heart, Lung, and Blood Institute’s recommended cut-off points [27]; FPG ≥100 mg/dL according to the recommendations of the American Diabetes Association [28]; fasting TG ≥110 mg/dL; and HDL-C < 40 mg/dL [29].

The second definition was based on a homeostatic model assessment of insulin resistance (HOMA-IR), using the cut-point proposed by Prince et al., by which participants with a HOMA-IR score ≥ 3.16 were deemed MUO [30].

### Statistical analysis

All statistical analyses were conducted using the Statistical Package for Social Sciences version 15.0 (SPSS Inc., Chicago, IL, USA). Normality of distribution was assessed using histogram charts and Kolmogorov–Smirnov analysis. Characteristics of participants were expressed as mean ± SD or median and interquartile range (IQR) for normal and skewed distributions, respectively, and percentages for categorical variables. Linear regression and chi-square analyses were used to test the trend of continuous and categorical variables, respectively, across tertiles of the DASH score.

To examine the association of obesity phenotypes in each tertile of the DASH score, logistic regression models were used; odds ratios (ORs) and 95% confidence intervals (CIs) were reported. In this analysis, in addition to the crude model, the confounding effects of sex, puberty status, physical activity, BMI Z-score, and passive smoking were controlled in model 2, and further adjustments for the intakes of energy, saturated fatty acids, and monounsaturated fatty acids were performed in model 3. *P*-values < 0.05 were considered significant.

## Results

The participants in this study (52.6% boys) had a mean ± SD of 9.3 ± 1.7 years for age and 24.0 ± 4.9 for the DASH score, with 17% being in the pre-pubertal stage. Moreover, 31% of the participants were categorised as overweight and 69% as obese. The prevalence of MUO was 62.2% based on RFs, and 43.4% based on HOMA-IR. Characteristics of children and adolescents according to tertiles of the DASH score are shown in Table 1. Participants in the third tertile of the DASH score had lower FPG than those in the first one (*P* for trend = 0.005). However, the other characteristics showed no significant trends across tertiles of the DASH score.

**Table 1:** . Characteristics of participants according to tertiles of the Dietary Approaches to Stop Hypertension (DASH) score.

	Tertiles of the DASH score			*P* for trend*
	T1 (*n* = 144)	T2 (*n* = 93)	T3 (*n* = 104)	
Age (y)	9.6 ± 1.8	9.0 ± 1.7	9.2 ± 1.6	0.058
Boys (%)	57.6	49.5	48.1	0.124
Obesity (%)	66.0	72.0	70.2	0.441
Body mass index Z-score	2.6 ± 0.7	2.6 ± 0.7	2.5 ± 0.6	0.482
Waist circumference (cm)	82.3 ± 10.0	80.4 ± 9.6	79.6 ± 8.8	0.053
Fasting plasma glucose (mg/dL)	92.0 ± 8.9	99.8 ± 9.1	88.9 ± 8.8	**0.005**
High-density lipoprotein cholesterol (mg/dL)	49.1 ± 12.0	50.5 ± 12.3	49.8 ± 11.2	0.686
Insulin (mIU/L)	13.6 (9.9–19.5)	12.1 (8.2–18.5)	11.0 (7.3–17.7)	0.566
Systolic blood pressure (mmHg)	105.0 (95.0–115.0)	105.0 (91.5–115.0)	105.0 (98.5–115.0)	0.316
Diastolic blood pressure (mmHg)	63.0 (60.0–70.0)	70.0 (60.0–74.0)	60.0 (60.0–70.0)	0.652
Triglycerides (mg/dL)	106.5 (75.2–138.8)	105.0 (75.5–148.0)	103.0 (76.0–140.8)	0.402
Prepuberty status (%)	19.4	18.3	12.5	0.163
Physical activity (MET hr./wk)	8.9 (2.5–23.9)	7.0 (1.6–19.5)	9.6 (3.0–24.2)	0.862
Passive smoking (%)	31.3	31.2	22.1	0.132

Data represented as mean ± SD or median (IQR 25–75) unless otherwise noted.

*Linear regression and Chi-square analyses were used to test the trend of continuous and categorical variables, respectively, according to tertiles of the DASH score

Participants in the highest tertile of the DASH score had lower intakes of energy and total fat compared to those in the lowest. In contrast, consumption of potassium, magnesium, calcium, protein, and carbohydrate increased across tertiles of the DASH-style diet. Moreover, the DASH components per 1000 kcal intake across tertiles of the DASH score showed an increasing trend for whole grains, vegetables, fruits, low-fat dairy, and nuts and seeds and legumes, and a decreasing trend for red and processed meat, sweetened beverages, and sodium (Table 2).

**Table 2:** Dietary intakes of participants according to tertiles of the Dietary Approaches to Stop Hypertension (DASH) score.

	Tertiles of the DASH score			*P* value*
	T1	T2	T3	
Daily intakes				
Components of DASH score (median score)	20	25	29	
Total energy (Kcal)	3021 ± 971	2757 ± 858	2688 ± 752	0.007
Dietary fiber (g/1000 Kcal)	16.6 ± 6.6	17.2 ± 5.4	18.9 ± 5.2	0.019
Added sugar (g/1000 Kcal)	20.2 ± 7.9	18.6 ± 8.7	16.5 ± 7.2	< 0.001
Potassium (mg/1000 Kcal)	1347 ± 237	1500 ± 273	1713 ± 329	< 0.001
Magnesium (mg/1000 Kcal)	142 ± 21	157 ± 29	171 ± 26	< 0.001
Calcium (mg/1000 Kcal)	449 ± 119	512 ± 125	536 ± 139	< 0.001
Protein (% energy)	13.2 ± 2.2	13.4 ± 2.2	13.6 ± 2.0	0.014
Carbohydrate (% energy)	54.6 ± 5.9	55.5 ± 5.4	58.5 ± 4.8	< 0.001
Total fat (% energy)	34.1 ± 5.8	33.6 ± 5.3	31.0 ± 4.6	< 0.001
Saturated fatty acids (% energy)	10.6 ± 2.5	10.4 ± 2.5	9.5 ± 1.8	< 0.001
Monounsaturated fatty acids (% energy)	10.9 ± 2.5	10.6 ± 2.3	9.7 ± 1.8	0.001
Polyunsaturated fatty acids (% energy)	7.1 ± 2.1	7.2 ± 2.2	6.7 ± 1.7	0.431
DASH components				
Whole grains (serving/1000 Kcal)	0.31 ± 0.47	0.46 ± 1.00	0.48 ± 0.50	0.026
Vegetable (serving/1000 Kcal)	0.73 ± 0.38	0.90 ± 0.42	1.23 ± 0.57	< 0.001
Fruits (serving/1000 Kcal)	0.81 ± 0.37	1.10 ± 0.60	1.49 ± 0.66	< 0.001
Low fat dairy (serving/1000 Kcal)	0.30 ± 0.30	0.50 ± 0.34	0.64 ± 0.40	< 0.001
Nuts, seeds, and legumes (serving/1000 Kcal)	0.49 ± 0.35	0.64 ± 0.40	0.81 ± 0.55	< 0.001
Red and processed meat (serving/1000 Kcal)	0.46 ± 0.27	0.32 ± 0.23	0.23 ± 0.16	< 0.001
Sweetened beverage (serving/1000 Kcal)	0.20 ± 0.22	0.12 ± 0.14	0.06 ± 0.10	< 0.001
Sodium (milligram/1000 Kcal)	1505 ± 373	1417 ± 513	1296 ± 363	< 0.001

Data represented as mean ± SD.

*Linear regression analysis was used to test the trend of continuous variables according to tertiles of the DASH score

Multivariable-adjusted ORs for MUO phenotypes across tertiles of the DASH score are presented in Table 3. Better adherence to the DASH-style diet was inversely related to the odds for MUO based on HOMA-IR classification. In the crude model, the association was insignificant (OR = 0.62; 95% CI: 0.37–1.03), but after adjustment for potential confounders, participants in the highest tertile of the DASH score had statistically significant decreased odds for MUO based on HOMA-IR definition (OR = 0.49; 95% CI: 0.28–0.87) compared with those in the lowest tertile. No associations were found between the DASH score and MUO based on RFs (OR = 0.68; 95% CI: 0.38–1.20), high blood pressure (OR = 1.20; 95% CI: 0.60–2.41), high TG (OR = 0.94; 95% CI: 0.54–1.62), low HDL-C (OR = 0.90; 95% CI: 0.44–1.81), high FPG (OR = 0.57; 95% CI: 0.27–1.19), and abdominal obesity (OR = 0.43; 95% CI: 0.13–1.41).

**Table 3:** Odds ratios (95% CI) for MUO phenotype and cardio-metabolic risk factors according to tertiles of the DASH score

Obesity Phenotype or Risk Factor	Tertiles of the DASH score			*P* for trend*
	T1 (*n* = 144)	T2 (*n* = 93)	T3 (*n* = 104)	
Median	20	25	29	
MUO based on risk factors				
Cases (%)	94 (65.3)	56 (60.2)	62 (59.6)	
Model 1	Ref	0.80 (0.47–1.38)	0.78 (0.47–1.32)	0.336
Model 2	Ref	0.81 (0.46–1.41)	0.72 (0.42–1.25)	0.235
Model 3	Ref	0.79 (0.45–1.39)	0.68 (0.38–1.20)	0.180
MUO based on HOMA-IR				
Cases (%)	71 (49.3)	38 (40.9)	39 (37.5)	
Model 1	Ref	0.71 (0.42–1.20)	0.62 (0.37–1.03)	0.056
Model 2	Ref	0.66 (0.38–1.15)	0.51 (0.30–0.88)	**0.013**
Model 3	Ref	0.66 (0.37–1.15)	0.49 (0.28–0.87)	**0.013**
High blood pressure				
Cases (%)	29 (20.3)	26 (28.3)	25 (24.0)	
Model 1	Ref	1.55 (0.84–2.85)	1.24 (0.68–2.28)	0.394
Model 2	Ref	1.89 (0.97–3.69)	1.35 (0.70–2.62)	0.271
Model 3	Ref	1.79 (0.91–3.52)	1.20 (0.60–2.41)	0.467
High triglycerides				
Cases (%)	66 (45.8)	43 (46.2)	47 (45.2)	
Model 1	Ref	1.02 (0.60–1.72)	0.97 (0.59–1.62)	0.934
Model 2	Ref	0.97 (0.56–1.66)	0.90 (0.53–1.52)	0.694
Model 3	Ref	1.00 (0.58–1.72)	0.94 (0.54–1.62)	0.823
Low HDL-C				
Cases (%)	30 (20.8)	21 (22.6)	17 (16.3)	
Model 1	Ref	1.11 (0.59–2.08)	0.74 (0.38–1.43)	0.449
Model 2	Ref	1.10 (0.58–2.08)	0.75 (0.39–1.47)	0.479
Model 3	Ref	1.20 (0.63–2.31)	0.90 (0.44–1.81)	0.874
High fasting blood glucose				
Cases (%)	31 (21.5)	15 (16.1)	14 (13.5)	
Model 1	Ref	0.70 (0.36–1.38)	0.57 (0.28–1.13)	0.093
Model 2	Ref	0.68 (0.34–1.37)	0.57 (0.28–1.15)	0.099
Model 3	Ref	0.70 (0.35–1.41)	0.57 (0.27–1.19)	0.116
Abdominal obesity				
Cases (%)	132 (92.3)	83 (93.3)	94 (92.2)	
Model 1	Ref	1.15 (0.41–3.24)	0.98 (0.38–2.53)	0.999
Model 2	Ref	1.03 (0.33–3.23)	0.70 (0.24–1.98)	0.534
Model 3	Ref	0.90 (0.28–2.94)	0.43 (0.13–1.41)	0.201

*CI* confidence interval, *MUO* metabolically unhealthy overweight/obese, *DASH *Dietary Approaches to Stop Hypertension, *HOMA-IR* homeostatic model assessment of insulin resistance, *HDL-C* high-density lipoprotein cholesterol

Model 1: Crude

Model 2: Adjusted for sex, puberty status, physical activity, BMI z-score, and passive smoking

Model 3: Adjusted for sex, puberty status, physical activity, BMI z-score, passive smoking, energy intake, saturated fatty acids intake, and monounsaturated fatty acids intake

*Based on logistic regression model using median scores of the DASH-style diet in each tertile as a continuous variable

## Discussion

In this sample of Tehranian children and adolescents with excess weight, the DASH-style diet was favourably associated with IR. To be more specific, better adherence to the DASH diet decreased the odds of MUO based on HOMA-IR definition by 51% when potentially confounding factors were adjusted. Interestingly, no significant association was observed between the DASH score and obesity phenotypes based on cardiometabolic RFs, or any of the RFs per se.

A previous national study reported the prevalence of obesity as 10.9%, MHO as 9.1%, and MUO as 1.8% among children and adolescents in Iran [6]. It means approximately 83.3 and 16.7% of children and adolescents affected by obesity were MHO and MUO, respectively, while in the current study, the prevalence of these phenotypes was 38.8 and 62.2%, respectively. However, in the aforementioned study, only obese participants but not overweight ones were included. Moreover, MHO was defined as the absence of MetS, which includes a higher proportion of the population compared to our definitions.

The DASH score is comprised of commonly used food groups in addition to sodium, which can provide a desirable tool for assessing whole dietary intake. Accordingly, the DASH score is capable of evaluating the quality of Iranian diets, even if the measure was developed for a Western dietary context. Previous studies of Iranian adolescents, measuring diet quality through the DASH score, have illustrated associations with the risk of MetS [12], and central and general obesity [13]. However, urban Iranian youth consume suboptimal amounts of whole grains, fruits, vegetables, legumes, nuts, seeds, and low-fat dairy, and undesirable amounts of refined grains, fast and processed food, sweetened beverages, and salty snacks [24]. In this study, meanwhile, a significant increasing trend for whole grains, vegetables, fruits, low-fat dairy, and nuts and seeds and legumes, in contrast to a significant decreasing trend for red and processed meat, sweetened beverages, and sodium were observed across tertiles of the DASH score. Therefore, the DASH score seems to successfully detect and evaluate variations in the current Iranian diet, which is the primary purpose of diet quality indices.

A few adult studies have assessed the association between the DASH score and obesity phenotypes [14, 15]. Within the framework of the NHANES III, among participants aged < 45 years, the DASH score showed no association with MHO phenotype but had an inverse association with metabolically obese normal weight (MONW) phenotype [14]. In another observational study carried out by Phillips et al. among Irish adults aged 45–74, better adherence to the DASH diet was likely to be associated with metabolic health in the non-obese participants, but not in the obese group [15]. The results of these studies may indicate that a DASH-style diet might prove to be more beneficial in non-obese adults compared to the obese.

Some previous studies have observed associations between unhealthy dietary patterns and IR in children and adolescents [31, 32]. Karatzi et al. observed a positive association between a dietary pattern characterised by high consumption of margarines, sweets and snacks, and HOMA-IR [31]. Furthermore, Appannah et al. reported that the pattern identified as energetically dense, high in fat, and low in fiber was associated with cardiometabolic alterations such as high insulin concentrations and IR [32]. Both aforementioned dietary patterns consist of items that are minimised in our DASH score.

In the current study, the DASH score was not associated with MUO based on cardiometabolic RFs, or any of the RFs per se. However, these outcomes are contradictory to the findings of some previous studies which have assessed the relationship between the DASH diet, MetS, and cardiometabolic markers in children and adolescents [12, 13]. It was previously observed that a DASH-style diet was inversely associated with the incidence of MetS, hypertension, high fasting plasma glucose, and abdominal obesity in participants aged 6–18 years [12], whereas in another study, higher DASH scores were associated with decreased risk of central and general obesity, but not associated with the risk of dyslipidaemia in adolescents [13]. Some probable explanations for these contradictory findings are as follows. The participants in the current study were all overweight and obese, whereas, in the two aforementioned ones, healthy at-risk samples were selected from a representative urban population. Moreover, the longitudinal design of these two studies might be the reason they were able to find associations, whereas the current study has a cross-sectional design.

Although a direct linkage between the DASH diet and IR is yet to be established, some mechanisms may explain its beneficial effects. A relationship between inflammation and IR in adolescents has previously been observed, which was particularly strong in those affected by obesity [33, 34]. A DASH-style diet is rich in whole grains, fruits, vegetables, nuts, and seeds; therefore, having high amounts of antioxidants, fiber, and magnesium, all of which may attenuate inflammation [35]. Apart from inflammation, whole grains have a low glycaemic index and are absorbed slowly; thus, leading to a slower rise in blood glucose and insulin levels [36]. Moreover, dietary fiber per se, which is abundant in the DASH diet, may have beneficial effects on fasting serum glucose, insulin levels, and IR in adolescents [37–39]. Furthermore, high intakes of calcium, potassium, and magnesium from a DASH-style diet may lead to better insulin sensitivity through multiple pathways [40].

The results of this study should be interpreted in light of several acknowledged limitations and strengths. One of the limitations is the cross-sectional design of the study, which does not allow any conclusion of causality. Moreover, measuring insulin sensitivity using the glucose clamp technique as the gold standard [41] would have resulted in more reliable data for our analyses. In addition, since the Iranian FCT is incomplete, we used the USDA FCT, which could result in some minor macro- and micronutrient miscalculations. Although we adjusted some of the most significant potential confounders, the socio-economic status of the participants, as an important confounder, was unavailable. Some strengths of the current study, on the other hand, include the presence of the participants’ mothers during face-to-face interviews, which made children more comfortable and was useful in case they had difficulty recalling or quantifying their diets. Moreover, all dietary and anthropometric assessments were performed by highly trained dietitians specialised in the paediatric field, which minimised errors in data collection.

## Conclusion

The DASH score was inversely associated with MUO based on IR in children and adolescents. However, no associations were found between the DASH score and MUO based on cardiometabolic RFs or any individual risk factor. A DASH-style diet may have favourable effects on insulin sensitivity among children and adolescents with excess weight. A dietary pattern resembling the DASH diet could be promoted in primary schools, especially to the students with excess weight. Our findings provide possibilities and hypotheses for future studies in this setting, which should obviate the limitations of our study and try longitudinal design to further improve our knowledge in this field and possibly find ways to halt the transition from MHO to MUO. However, universal definitions for obesity phenotypes are required.

## Data Availability

The authors of this study commit to making requested and de-identified participant data available after reasonable request, up until 5 years after publication of the manuscript. Requests for access to data require evidence of ethics approval of a methodologically sound proposal for use, and data sharing agreements must be in place. Requests should be addressed to the corresponding author at g_asghari@hotmail.com.

## Abbreviations

DASH: Dietary approaches to stop hypertension
MHO: Metabolically healthy overweight/obese
MUO: Metabolically unhealthy overweight/obese
RFs: Risk factors
IR: Insulin resistance
HOMA-IR: Homeostatic model assessment of insulin resistance
MetS: Metabolic syndrome
BMI: Body mass index
WC: Waist circumference
FPG: Fasting plasma glucose
TG: Triglycerides
HDL-C: High-density lipoprotein cholesterol
